# Supplementary material for: Cord blood DNA methylome in newborns later diagnosed with autism spectrum disorder reflects early dysregulation of neurodevelopmental and X-linked genes
Source: Genome Med. 2020 Oct 14;12:88. doi: 10.1186/s13073-020-00785-8 (PMC7559201; doi:10.1186/s13073-020-00785-8)
Supplement: Supplementary file 1 — Additional file 1: Figure S1. Global CpG methylation is associated with diagnosis and behavioral outcome scores only in males. Figure S2. The proportion of nRBCs is associated with behavioral outcome and global CpG methylation in males. Figure S3. Methylation at ASD DMRs in the discovery set is specifically associated with behavioral outcome. Figure S4. DMRs identified in all discovery subjects distinguish ASD from TD subjects. Figure S5. Sequencing platform has a larger effect on 10 kb window methylation than ASD diagnosis, sex, or study. Figure S6. DMRs identified in males and females distinguish ASD from TD subjects in the replication set. Figure S7. Methylation at ASD DMRs in the replication set is specifically associated with behavioral outcome. Figure S8. DMRs identified in all replication subjects distinguish ASD from TD subjects. Figure S9. The majority of ASD DMRs do not overlap probes on the 450 K and EPIC arrays. Figure S10. DMR methylation assayed by WGBS correlates with DMR methylation assayed by bisulfite pyrosequencing. Figure S11. A subset of ASD diagnosis DMRs are associated with ASD severity in independent sample sets. Figure S12. Machine learning methods workflow. Figure S13. Summary of machine learning datasets and results for males. Figure S14. Summary of machine learning datasets and results for females. Figure S15. ASD DMRs identified with adjustment for sex miss many genes found when stratifying for sex. Figure S16. Replicated ASD DMB genes overlap with replicated ASD DMR genes. Figure S17. ASD DMB genes are enriched for membrane, cell adhesion, and embryo-expressed genes. Figure S18. Neurodevelopmental genes are overrepresented on the X chromosome. Figure S19. Replicated DMR genes on the X chromosome are expressed in fetal brain. Figure S20. Female-specific replicated DMR genes on the X chromosome are expressed in fetal brain. Figure S21. Cord blood ASD DMR genes are significantly enriched for epigenetically dysregulated genes in ASD brain. [file 13073_2020_785_MOESM1_ESM.docx]

**Additional File 1: Supplemental Figures**

**Table of Contents**

| **Lower global DNA methylation in umbilical cord blood from males later diagnosed with ASD corresponds with increased nucleated red blood cells** | **3** |
| --- | --- |
| Fig. S1. Global CpG methylation is associated with diagnosis and behavioral outcome scores only in males | 3 |
| Fig. S2. The proportion of nRBCs is associated with behavioral outcome and global CpG methylation in males | 4 |
| **Region-specific differential methylation patterns in umbilical cord blood distinguish males and females later diagnosed with ASD from typically developing controls** | **6** |
| Fig. S3. Methylation at ASD DMRs in the discovery set is specifically associated with behavioral outcome | 6 |
| Fig. S4. DMRs identified in all discovery subjects distinguish ASD from TD subjects | 8 |
| Fig. S5. Sequencing platform has a larger effect on 10 kb window methylation than ASD diagnosis, sex, or study | 10 |
| Fig. S6. DMRs identified in males and females distinguish ASD from TD subjects in the replication set | 11 |
| Fig. S7. Methylation at ASD DMRs in the replication set is specifically associated with behavioral outcome | 12 |
| Fig. S8. DMRs identified in all replication subjects distinguish ASD from TD subjects | 14 |
| Fig. S9. The majority of ASD DMRs do not overlap probes on the 450K and EPIC arrays | 15 |
| Fig. S10. DMR methylation assayed by WGBS correlates with DMR methylation assayed by bisulfite pyrosequencing | 16 |
| **ASD DMRs in umbilical cord blood replicate across independent groups of subjects** | **17** |
| Fig. S11. A subset of ASD diagnosis DMRs are associated with ASD severity in independent sample sets | 17 |
| Fig. S12. Machine learning methods workflow | 18 |
| Fig. S13. Summary of machine learning datasets and results for males | 19 |
| Fig. S14. Summary of machine learning datasets and results for females | 20 |
| Fig. S15. ASD DMRs identified with adjustment for sex miss many genes found when stratifying for sex | 21 |
| **Genes in ASD differentially-methylated blocks replicate between independent groups of subjects and are enriched for ASD DMR genes, cadherins, and developmental genes** | **22** |
| Fig. S16. Replicated ASD DMB genes overlap with replicated ASD DMR genes | 22 |
| Fig. S17. ASD DMB genes are enriched for membrane, cell adhesion, and embryo-expressed genes | 23 |
| **Cord blood ASD DMR genes are enriched for neurodevelopmental genes on the X chromosome that are epigenetically dysregulated in ASD brain** | **24** |
| Fig. S18. Neurodevelopmental genes are overrepresented on the X chromosome | 24 |
| Fig. S19. Replicated DMR genes on the X chromosome are expressed in fetal brain | 25 |
| Fig. S20. Female-specific replicated DMR genes on the X chromosome are expressed in fetal brain | 26 |
| Fig. S21. Cord blood ASD DMR genes are significantly enriched for epigenetically dysregulated genes in ASD brain | 28 |
| Fig. S22. Selected regions with replicated sex-independent DMR genes on the X chromosome | 30 |
| Fig. S23. Selected regions with replicated male-specific DMR genes on the X chromosome | 31 |
| Fig. S24. Selected regions with replicated female-specific DMR genes on the X chromosome | 32 |
| **ASD DMRs are enriched for a pan-tissue epigenomic signature that differs between males and females on the X chromosome** | **33** |
| Fig. S25. ASD DMRs in replication subjects are differentially enriched for chromatin states on the X chromosome | 33 |
| Fig. S26. ASD DMRs in discovery subjects are differentially enriched for histone PTMs on the X chromosome | 35 |
| Fig. S27. ASD DMRs in replication subjects are differentially enriched for histone PTMs on the X chromosome | 36 |
| Fig. S28. ASD DMRs on the X chromosome are enriched near CpG islands only in females | 37 |

**Lower global DNA methylation in umbilical cord blood from males later diagnosed with ASD corresponds with increased nucleated red blood cells**

**Fig. S1. Global CpG methylation is associated with diagnosis and behavioral outcome scores only in males.** (A) Estimated change in global methylation with behavioral, demographic, and technical variables. *P*-values were adjusted for the number of variables using the false discovery rate (FDR) method (* *q* < 0.05). (B) Global CpG methylation compared to Mullen Early Learning Composite score is plotted by sex and sample set (* *p* < 0.05, pooled males *p* = 7.9E-4, pooled females *p* = 0.96). (A,B) Significance was tested using linear regression with adjustment for PCR duplicates, and also adjusted for sequencing platform when pooled (pooled males typically developing (TD) *n* = 56, autism spectrum disorder (ASD) *n* = 56; pooled females TD *n* = 20, ASD *n* = 20). ADOS, Autism Diagnostic Observation Schedule; BMI, body mass index; NK, natural killer; nRBC, nucleated red blood cell;

**Fig. S2. The proportion of nRBCs is associated with behavioral outcome and global CpG methylation in males.** (A) Percent CpG methylation across the genome. Boxes represent mean and 95% confidence limits by nonparametric bootstrapping. Linear model included adjustment for PCR duplicates (pooled males *p* = 0.002, pooled females *p* = 0.70). (B) Estimated proportion of nRBCs. Boxes represent mean and 95% confidence limits by nonparametric bootstrapping (pooled males *p* = 0.003, pooled females *p* = 0.78). (C) Estimated proportion of nRBCs compared to Mullen Early Learning Composite score is plotted by sex and sample set (pooled males *p* = 0.003, pooled females *p* = 0.71). (D) Estimated proportion of nRBCs compared to global CpG methylation is plotted by sex and sample set (pooled males *p* = 1.7E-18, pooled females *p* = 3.1E-5). Significance was tested using linear regression with adjustment for sequencing platform when pooled (* *p* < 0.05; pooled males TD *n* = 56, ASD *n* = 56; pooled females TD *n* = 20, ASD *n* = 20).

**Region-specific differential methylation patterns in umbilical cord blood distinguish males and females later diagnosed with ASD from typically developing controls**

**Fig. S3. Methylation at ASD DMRs in the discovery set is specifically associated with behavioral outcome.** Raw percent methylation at ASD differentially-methylated regions (DMRs) identified in (A) male or (B) female discovery set subjects was compared with demographic and technical variables. Significance testing was done with linear regression and the -log_10_(p-value) was plotted (males TD *n* = 39, ASD *n* = 35; females TD *n* = 17, ASD *n* = 15). Edu, education;

**Fig. S4. DMRs identified in all discovery subjects distinguish ASD from TD subjects.** (A) Heatmap or (B) principal component analysis (PCA) plot using percent methylation for each sample at ASD DMRs identified in all discovery subjects with adjustment for sex (42 hypermethylated DMRs, 145 hypomethylated DMRs). For heatmap, subjects are colored by diagnostic group and study, and methylation is relative to the mean for each DMR. For PCA plot, ellipses indicate 95% confidence limits. (C) Raw percent methylation at ASD DMRs identified in all discovery subjects was compared with demographic and technical variables. Significance testing was done using linear regression with adjustment for sex and the -log_10_(p-value) was plotted (males TD *n* = 39, ASD *n* = 35; females TD *n* = 17, ASD *n* = 15). EARLI, Early Autism Risk Longitudinal Investigation; F, female; M, male; MARBLES, Markers of Autism Risk in Babies - Learning Early Signs;

**Fig. S5. Sequencing platform has a larger effect on 10 kb window methylation than ASD diagnosis, sex, or study.** PCA plots using percent methylation at 10 kb windows tiled across the genome for each sample. Points were colored by ASD diagnosis, sex, platform, or study. Ellipses indicate 95% confidence limits (males TD *n* = 56, ASD *n* = 56; females TD *n* = 20, ASD *n* = 20).

**Fig. S6. DMRs identified in males and females distinguish ASD from TD subjects in the replication set.** (A) Heatmap or (B) PCA plot using percent methylation for each sample at ASD DMRs identified in male replication subjects (975 hypermethylated DMRs, 3675 hypomethylated DMRs). (C) Heatmap or (D) PCA plot using percent methylation for each sample at ASD DMRs identified in female replication subjects (4232 hypermethylated DMRs, 4496 hypomethylated DMRs). For heatmaps, subjects are colored by diagnostic group and study, and methylation is relative to the mean for each DMR. For PCA plots, ellipses indicate 95% confidence limits (males TD *n* = 17, ASD *n* = 21; females TD *n* = 3, ASD *n* = 5).

**Fig. S7. Methylation at ASD DMRs in the replication set is specifically associated with behavioral outcome.** Raw percent methylation at ASD DMRs identified in (A) male or (B) female replication set subjects was compared with demographic and technical variables. Significance testing was done with linear regression and the -log_10_(p-value) was plotted (males TD *n* = 17, ASD *n* = 21; females TD *n* = 3, ASD *n* = 5).

**Fig. S8. DMRs identified in all replication subjects distinguish ASD from TD subjects.** (A) Heatmap or (B) PCA plot using percent methylation for each sample at ASD DMRs identified in all replication subjects with adjustment for sex (614 hypermethylated DMRs, 3207 hypomethylated DMRs). For heatmap, subjects are colored by diagnostic group and study, and methylation is relative to the mean for each DMR. For PCA plot, ellipses indicate 95% confidence limits. (C) Raw percent methylation at ASD DMRs identified in all replication subjects was compared with demographic and technical variables. Significance testing was done using linear regression with adjustment for sex and the -log_10_(p-value) was plotted (males TD *n* = 17, ASD *n* = 21; females TD *n* = 3, ASD *n* = 5).


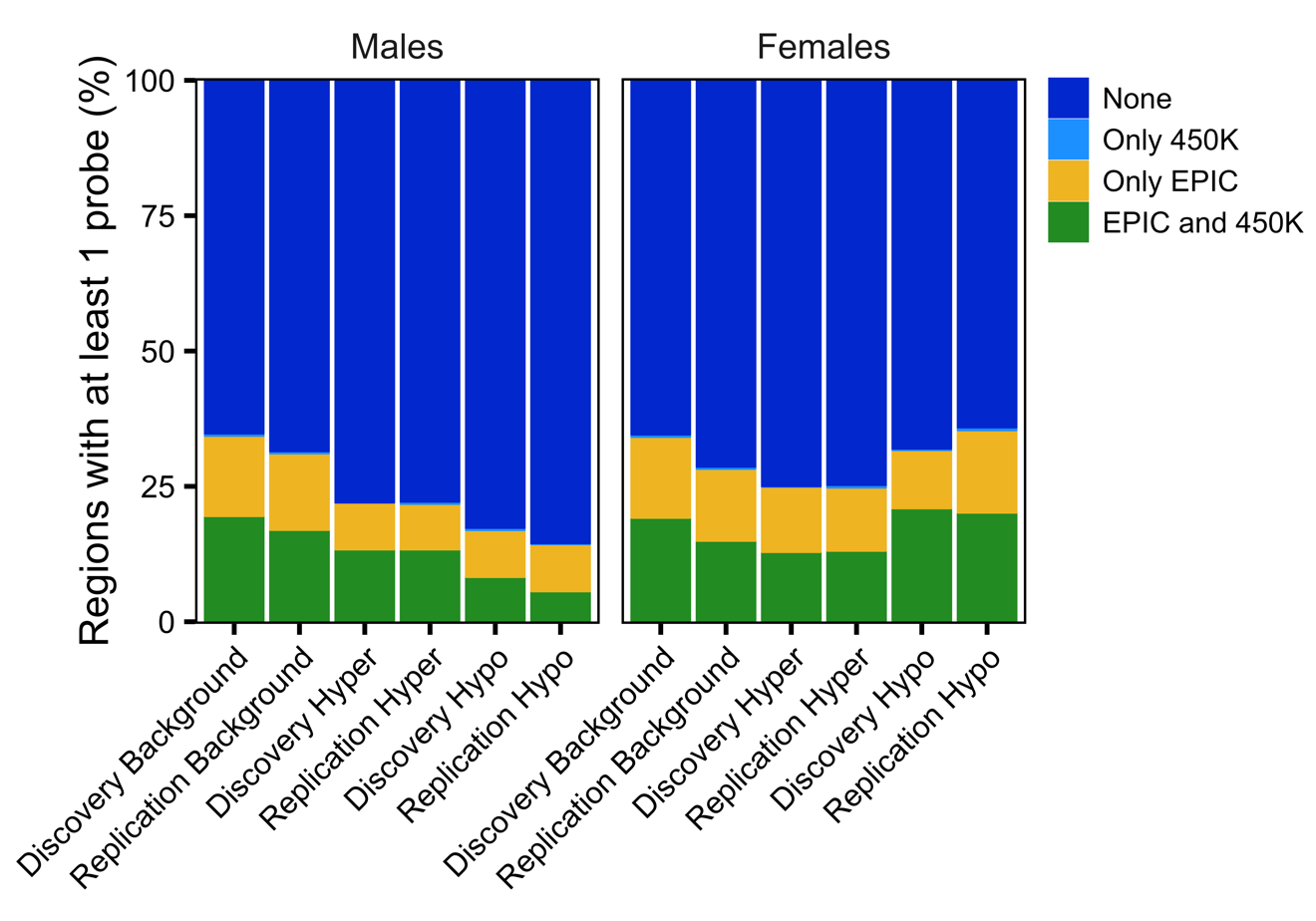


**Fig. S9. The majority of ASD DMRs do not overlap probes on the 450K and EPIC arrays.** ASD DMRs and background regions in Discovery or Replication Male or Female subjects were overlapped by location with probes on the Infinium HumanMethylation450 (450K) and MethylationEPIC (EPIC) arrays, and the proportion of regions overlapping at least one probe on either or both arrays was plotted (pooled males TD *n* = 56, ASD *n* = 56; pooled females TD *n* = 20, ASD *n* = 20). Hyper, hypermethylated; Hypo, hypomethylated;

**Fig. S10. DMR methylation assayed by WGBS correlates with DMR methylation assayed by bisulfite pyrosequencing.** Pyrosequencing assays were designed for selected ASD DMRs from the males replication comparison and conducted in a subset of male samples. Mean methylation values from bisulfite pyrosequencing were compared against those from the same CpG sites assayed with WGBS and tested using Pearson’s r (* *q* < 0.05, TD *n* = 10, ASD *n* = 12).

**ASD DMRs in umbilical cord blood replicate across independent groups of subjects**

**Fig. S11. A subset of ASD diagnosis DMRs are associated with ASD severity in independent sample sets.** (A,C) Volcano plots of the association between Discovery DMR methylation and ADOS comparison score for (A) ASD males or (C) ASD females by Discovery or Replication sample set. The x-axis represents the change in percent methylation per unit of the ADOS comparison score. (B,D-F) Scatterplots of ADOS comparison score versus percent methylation at Discovery DMRs with nominal significance (*p* < 0.05) in both Discovery and Replication sample sets in (B) ASD males or (D-F) ASD females (ASD males: Discovery *n* = 35, Replication *n* = 21; ASD females: Discovery *n* = 15, Replication *n* = 5).

Fig. S12. Machine learning methods workflow. Two datasets of smoothed discovery DMR methylation for samples in the discovery and replication sets were batch corrected with the ComBat() function from the sva R package. The final training set consisted of the batch-adjusted smoothed methylation values for samples in the discovery set. The final testing set consisted of the batch-adjusted smoothed methylation values for samples in the replication set. Using the caret R package, k-fold cross validation with the random forest model was applied on the training set three times, each with different mtry values. The random forest model with mtry value that resulted in the best training model performance was selected. The model was tested on the testing set to obtain the confusion matrix and performance metrics including observed accuracy, kappa statistic, sensitivity, specificity, and balanced accuracy. The model was also used to obtain the most predictive DMR predictors using the caret feature selection function varImp().

**Fig. S13. Summary of machine learning datasets and results for males**. (A) Summary of training set components. (B) Summary of testing set components. (C) Machine learning classification confusion matrix. (D) Machine learning classification performance metrics.

**Fig. S14. Summary of machine learning datasets and results for females.** (A) Summary of training set components. (B) Summary of testing set components. (C) Machine learning classification confusion matrix. (D) Machine learning classification performance metrics.

**Fig. S15. ASD DMRs identified with adjustment for sex miss many genes found when stratifying for sex.** (A) Venn diagram of genes annotated to ASD DMRs with adjustment for sex in all samples in Discovery and Replication sets. Significance was tested using the hypergeometric test and was relative to genes annotated to background regions. (B) Overlap of replicated ASD DMR genes between all samples, males, and females (pooled males TD *n* = 56, ASD *n* = 56; pooled females TD *n* = 20, ASD *n* = 20). Adj, adjusted for; OR, odds ratio;

**Genes in ASD differentially-methylated blocks replicate between independent groups of subjects and are enriched for ASD DMR genes, cadherins, and developmental genes**

**Fig. S16. Replicated ASD DMB genes overlap with replicated ASD DMR genes.** Selected regions with replicated DMRs and differentially-methylated blocks (DMBs) in males or females. chr, chromosome;

**Fig. S17. ASD DMB genes are enriched for membrane, cell adhesion, and embryo-expressed genes.** Terms significantly enriched among ASD DMB genes in both discovery and replication sample sets for either males or females (* *q* < 0.05). Heatmaps show -log_10_(*q*-value) for enrichment in genes annotated to DMBs relative to genes annotated to background calculated using the Database for Annotation, Visualization, and Integrated Discovery (DAVID) for all categories. Terms were sorted by replication sex (pooled males TD *n* = 56, ASD *n* = 56; pooled females TD *n* = 20, ASD *n* = 20).

**Cord blood ASD DMR genes are enriched for neurodevelopmental genes on the X chromosome that are epigenetically dysregulated in ASD brain**


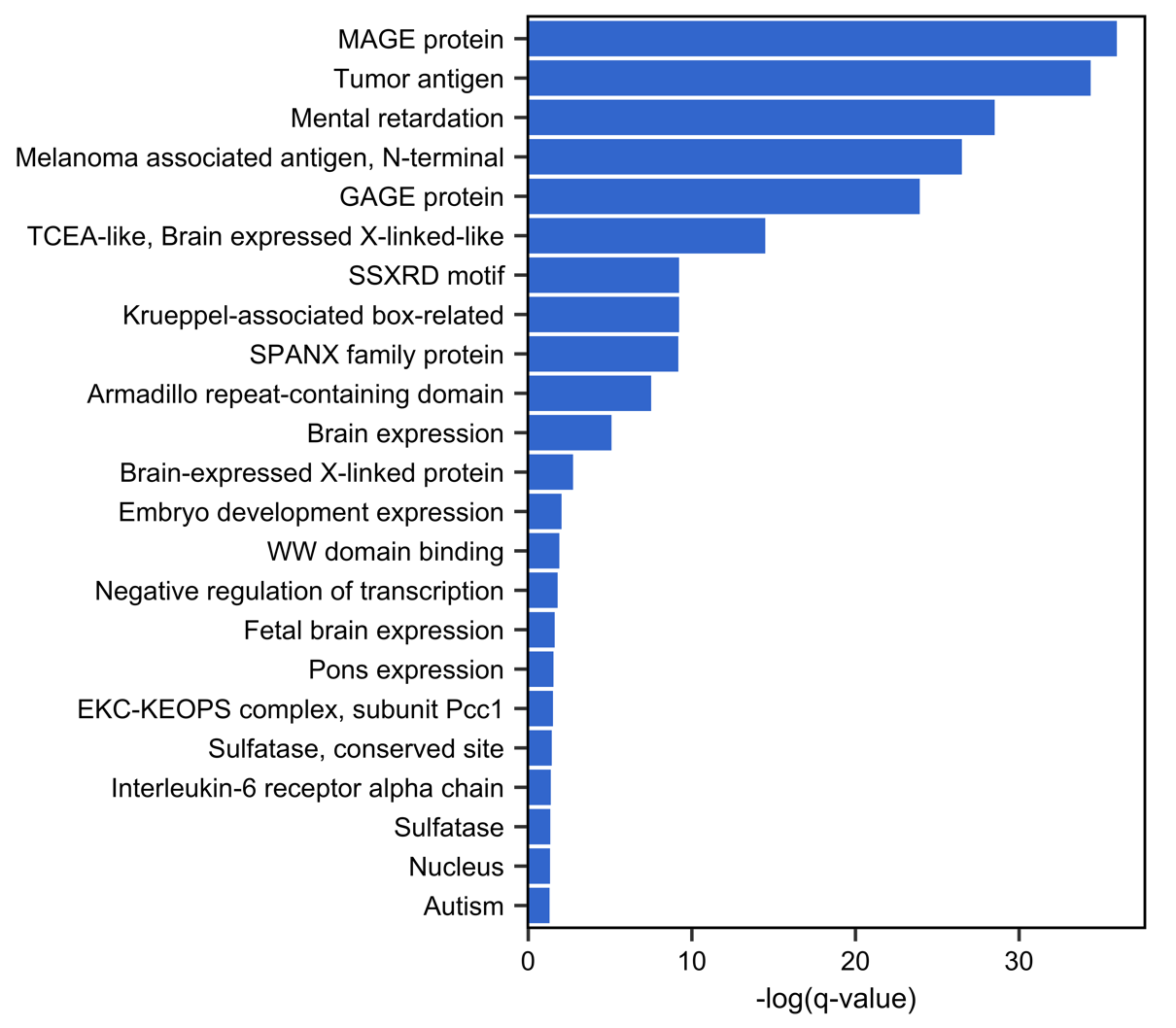


**Fig. S18. Neurodevelopmental genes are overrepresented on the X chromosome.** Terms significantly enriched among chromosome X genes compared to all genes (*q* < 0.05). Plot shows -log_10_(*q*-value) for enrichment calculated using DAVID for all categories.

**Fig. S19. Replicated DMR genes on the X chromosome are expressed in fetal brain.** RNA-seq expression values were obtained from the Allen BrainSpan Atlas of the Developing Human Brain for one male and one female dorsolateral prefrontal cortex at 13 weeks post-conception for X-linked DMR genes replicated in (A) males and females or (B) males only. RPKM, reads per kilobase per million reads;


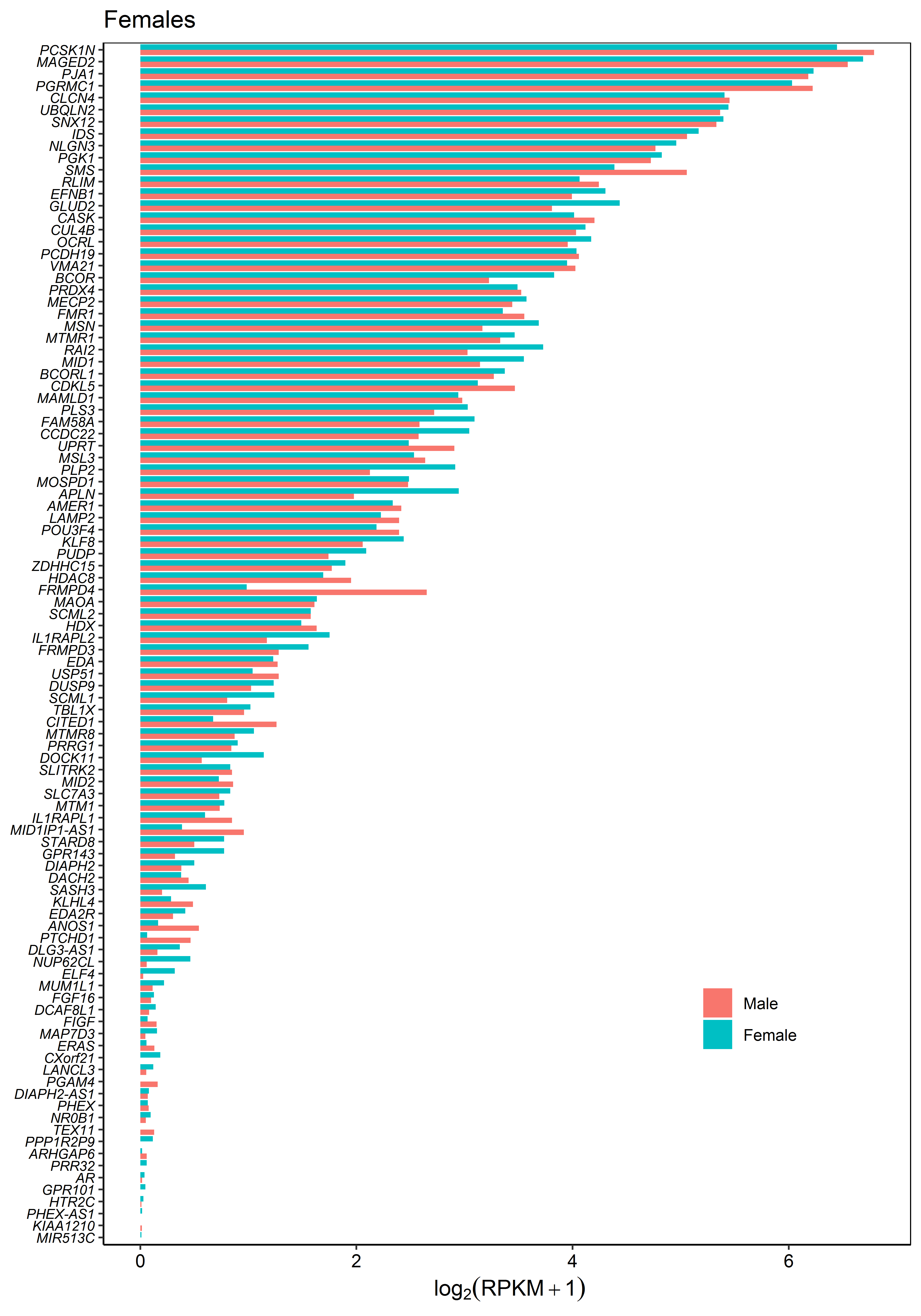


**Fig. S20. Female-specific replicated DMR genes on the X chromosome are expressed in fetal brain.** RNA-seq expression values were obtained from the Allen BrainSpan Atlas of the Developing Human Brain for one male and one female dorsolateral prefrontal cortex at 13 weeks post-conception for X-linked DMR genes replicated in females only.


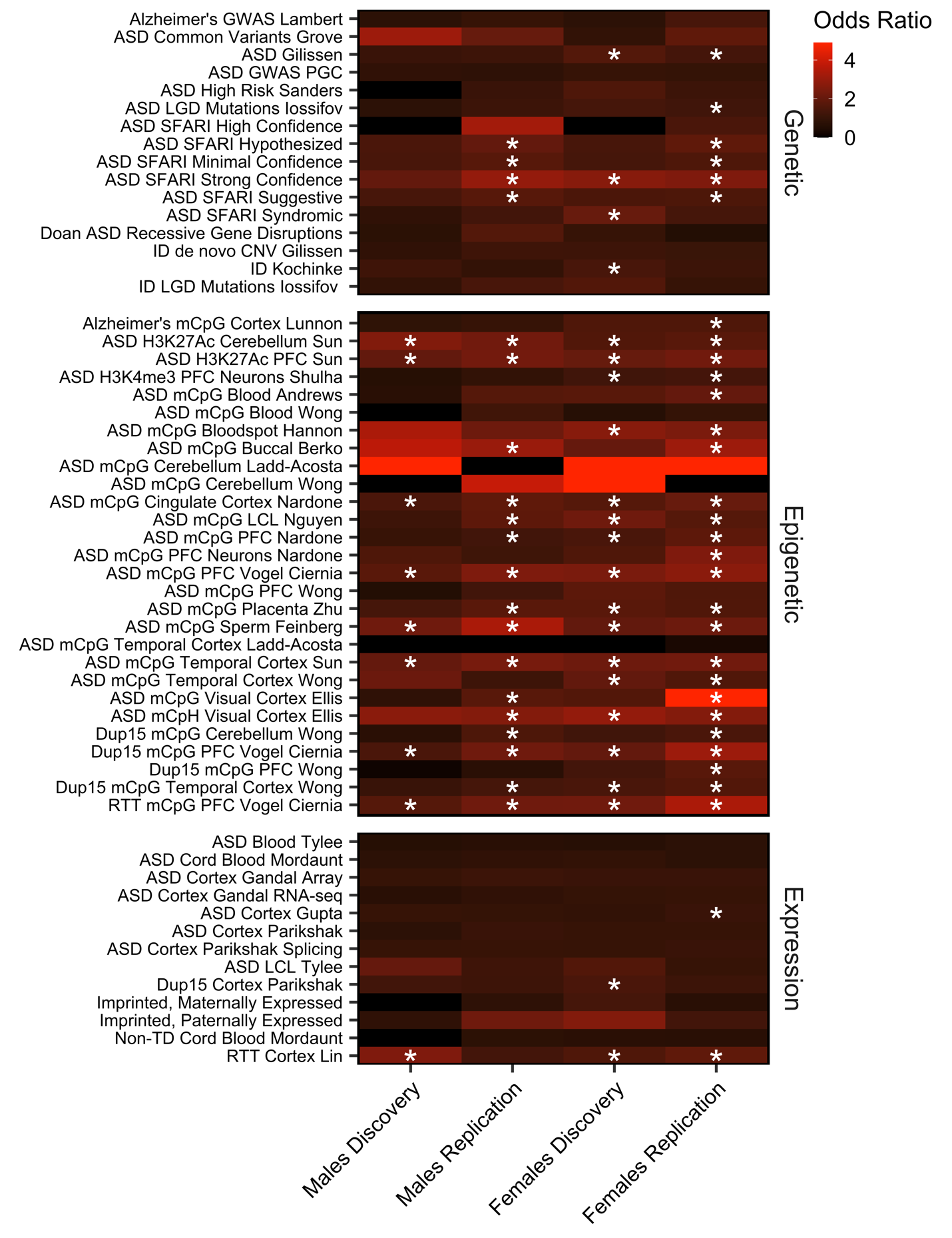


**Fig. S21. Cord blood ASD DMR genes are significantly enriched for epigenetically dysregulated genes in ASD brain.** All gene sets overlapped with ASD DMR genes in males or females (* *q* < 0.05). Heatmaps show odds ratios for enrichment in genes annotated to DMRs relative to genes annotated to background calculated with Fisher’s exact test for previously published studies of ASD and other neurological disorders. P-values were adjusted using the FDR method for the total number of gene lists compared (pooled males TD *n* = 56, ASD *n* = 56; pooled females TD *n* = 20, ASD *n* = 20). Ac, acetylation; Dup15, Chromosome 15q11-q13 Duplication syndrome; GWAS, genome wide association study; H3K27, histone 3 lysine 27; H3K4, histone 3 lysine 4; ID, intellectual disability; LCL, lymphoblastoid cell line; LGD, likely gene disrupting; mCpG, CpG methylation; mCpH, CpH methylation; me3, trimethylation; Non-TD, non-typically developing; PFC, prefrontal cortex; PGC, Psychiatric Genomics Consortium; RTT, Rett syndrome; SFARI, Simons Foundation Autism Research Initiative;

**Fig. S22. Selected regions with replicated sex-independent DMR genes on the X chromosome.** All shown regions replicated in males and females, were reported in a previous epigenetic study of ASD, and are expressed in fetal brain. Fetal brain ChromHMM chromatin state tracks were obtained from the Roadmap Epigenomics Project.

**Fig. S23. Selected regions with replicated male-specific DMR genes on the X chromosome.** All shown regions replicated in males only, were reported in a previous epigenetic study of ASD, and are expressed in fetal brain. Fetal brain ChromHMM chromatin state tracks were obtained from the Roadmap Epigenomics Project.

**Fig. S24. Selected regions with replicated female-specific DMR genes on the X chromosome.** All shown regions replicated in females only, were reported in a previous epigenetic study of ASD, and are expressed in fetal brain. Fetal brain ChromHMM chromatin state tracks were obtained from the Roadmap Epigenomics Project.

**ASD DMRs are enriched for a pan-tissue epigenomic signature that differs between males and females on the X chromosome**

**Fig. S25. ASD DMRs in replication subjects are differentially enriched for chromatin states on the X chromosome.** ASD DMRs were overlapped with 15-state model ChromHMM segmentations from 127 cell types in the Roadmap Epigenomics Project using the Locus Overlap Analysis (LOLA) R package. (A,C) The enrichment odds ratio was plotted for hypermethylated and hypomethylated DMRs identified in (A) males or (C) females from the replication set. Top enriched (•) chromatin states were identified as those with odds ratio and -log(*q*-value) of at least the median value for that DMR set and with *q* < 0.05 for more than half of all cell types. (B,D) The enrichment odds ratio was plotted for hypermethylated and hypomethylated DMRs on autosomes or the X chromosome identified in (B) males or (D) females from the replication set. Boxes represent mean and 95% confidence limits by nonparametric bootstrapping. Significance of differential enrichment of X chromosome compared to autosome DMRs was assessed by paired t-test of odds ratios for each cell type. P-values were adjusted for the number of chromatin states using the FDR method (* *q* < 0.05, males TD *n* = 17, ASD *n* = 21; females TD *n* = 3, ASD *n* = 5). BivFlnk, flanking bivalent transcription start site or enhancer; Enh, enhancer; EnhBiv, bivalent enhancer; EnhG, genic enhancer; Het, heterochromatin; Quies, quiescent region; ReprPC, polycomb-repressed region; ReprPCWk, weak polycomb-repressed region; TssA, active transcription start site; TssAFlnk, flanking active transcription start site; TssBiv, bivalent transcription start site; Tx, strong transcription; TxFlnk, transcribed at gene 5’ and 3’; TxWk, weak transcription; ZnfRpts, zinc finger genes and repeats;

**Fig. S26. ASD DMRs in discovery subjects are differentially enriched for histone PTMs on the X chromosome.** ASD DMRs were overlapped with histone post-translational modification (PTM) peaks from 127 cell types in the Roadmap Epigenomics Project using LOLA. (A,C) The enrichment odds ratio was plotted for hypermethylated and hypomethylated DMRs identified in (A) males or (C) females from the discovery set. Top enriched (•) histone PTMs were identified as those with odds ratio and -log(*q*-value) of at least the median value for that DMR set and with *q* < 0.05 for more than half of all cell types. (B,D) The enrichment odds ratio was plotted for hypermethylated and hypomethylated DMRs on autosomes or the X chromosome identified in (B) males or (D) females from the discovery set. Boxes represent mean and 95% confidence limits by nonparametric bootstrapping. Significance of differential enrichment of X chromosome compared to autosome DMRs was assessed by paired t-test of odds ratios for each cell type. P-values were adjusted for the number of histone PTMs using the FDR method (* *q* < 0.05, males TD *n* = 39, ASD *n* = 35; females TD *n* = 17, ASD *n* = 15). me1, monomethylation; H3K9, histone 3 lysine 9; H3K36, histone 3 lysine 36;

**Fig. S27. ASD DMRs in replication subjects are differentially enriched for histone PTMs on the X chromosome.** ASD DMRs were overlapped with histone PTM peaks from 127 cell types in the Roadmap Epigenomics Project using LOLA. (A,C) The enrichment odds ratio was plotted for hypermethylated and hypomethylated DMRs identified in (A) males or (C) females from the replication set. Top enriched (•) histone PTMs were identified as those with odds ratio and -log(*q*-value) of at least the median value for that DMR set and with *q* < 0.05 for more than half of all cell types. (B,D) The enrichment odds ratio was plotted for hypermethylated and hypomethylated DMRs on autosomes or the X chromosome identified in (B) males or (D) females from the replication set. Boxes represent mean and 95% confidence limits by nonparametric bootstrapping. Significance of differential enrichment of X chromosome compared to autosome DMRs was assessed by paired t-test of odds ratios for each cell type. P-values were adjusted for the number of histone PTMs using the FDR method (* *q* < 0.05, males TD *n* = 17, ASD *n* = 21; females TD *n* = 3, ASD *n* = 5).

**Fig. S28. ASD DMRs on the X chromosome are enriched near CpG islands only in females.** ASD DMRs and background regions on (A) autosomes or (B) chrX were intersected with CpG islands, shores, shelves, and open sea as defined in the annotatr R package, and the proportion of total basepairs in each of these contexts was plotted. ASD DMRs on (C) autosomes or (D) chrX were overlapped with CpG contexts using LOLA and the enrichment odds ratio relative to background regions was plotted (* *q* < 0.05, pooled males TD *n* = 56, ASD *n* = 56; pooled females TD *n* = 20, ASD *n* = 20).
